# Recent Advances in Stimuli-Sensitive Amphiphilic Polymer-Paclitaxel Prodrugs

**DOI:** 10.3389/fbioe.2022.875034

**Published:** 2022-04-06

**Authors:** Man Zhou, Lijuan Wen, Cui Wang, Qiao Lei, Yongxiu Li, Xiaoqing Yi

**Affiliations:** ^1^ College of Chemistry, Nanchang University, Nanchang, China; ^2^ College of Pharmacy, Gannan Medical University, Ganzhou, China; ^3^ School of Basic Medical Sciences, Anhui Medical University, Hefei, China

**Keywords:** paclitaxel, polymer prodrug, stimuli-sensitive, drug delivery, cancer

## Abstract

Paclitaxel (PTX) is a broad-spectrum chemotherapy drug employed in the treatment of a variety of tumors. However, the clinical applications of PTX are limited by its poor water solubility. Adjuvants are widely used to overcome this issue. However, these adjuvants often have side effects and poor biodistribution. The smart drug delivery system is a promising strategy for the improvement of solubility, permeability, and stability of drugs, and can promote sustained controlled release, increasing therapeutic efficacy and reducing side effects. Polymeric prodrugs show great advantages for drug delivery due to their high drug loading and stability. There has been some groundbreaking work in the development of PTX-based stimulus-sensitive polymeric prodrug micelles, which is summarized in this study. We consider these in terms of the four main types of stimulus (pH, reduction, enzyme, and reactive oxygen species (ROS)). The design, synthesis, and biomedical applications of stimulus-responsive polymeric prodrugs of PTX are reviewed, and the current research results and future directions of the field are summarized.

## Introduction

Paclitaxel (PTX) was first discovered in the late 1960s. It is a natural substance derived from the needles and bark of the Pacific yew tree. It is a mitotic inhibitor used primarily in cancer chemotherapy, which can be used in the treatment of various cancers, including ovarian cancer, esophagus cancer, small and non–small-cell lung cancer, multiple myeloma cancer, bladder cancer, breast cancer, head and neck cancer, colon cancer, and Kaposi’s sarcoma (Khalifa et al., 2019; Chen et al., 2021; Peng et al., 2021; Zuo et al., 2021). PTX is a microtubule stabilizing drug, which can prevent cytoskeletal microtubules from depolymerizing into free tubulin. It can also cause tubulin and tubulin dimer of microtubule to lose dynamic balance, leading to cell cycle arrest and stagnation in the G2/M phase, ultimately inhibiting cell growth (Ashrafizadeh et al., 2021; Mosca et al., 2021; Yi et al., 2022). The efficacy of PTX is primarily attributable to its side chain functionalities, A ring, oxetane ring, and C2 benzoyl group. The acyl group of the C3’ amide in the C13 chain remains active, and its function is enhanced by the hydroxyl group of C2’ (Zhang et al., 2013). PTX is insoluble in water (<0.3 mg/ml), and increasing its solubility by adjusting its pH is difficult. This greatly limits its clinical application. At present, the PTX dosage forms such as Taxol, PTX liposome, and PTX albumin binding are used in clinical treatment of breast cancer and ovarian cancer. However, the adjuvants in current clinical preparations have serious side effects. Moreover, it is difficult to achieve controlled drug release at the tumor site. Therefore, there is an urgent need to develop a novel drug delivery system that can improve the permeability and solubility of PTX and facilitate controlled targeted drug delivery, reducing side effects, and improving therapeutic efficacy.

In recent years, the use of self-assembling nanoparticles of amphiphilic polymers has become common in drug delivery as they are able to act as carriers for the hydrophobic molecules of insoluble drugs (Cabral and Kataoka, 2014; Ulbrich et al., 2016). Compared with traditional drug delivery systems, polymeric micelles have many merits such as the well-controlled size distribution, improved solubility, the enhanced permeability and retention (EPR) effect, good stability, and reduced toxicity (Chowdhury and Singh, 2020; Majumder et al., 2020). In addition, drug-targeting at the lesion site can be achieved by introducing targeted functional groups on the surfaces of the amphiphilic polymers (Roacho Perez et al., 2021). Amphiphilic polymers can load hydrophobic drugs through physical interaction or chemical bonding. Loading the drug to the polymers through physical interaction usually includes electrostatic interaction, π–π stacking, hydrogen bonding, and hydrophobic interaction (Zhai S. et al., 2017; Gao C. et al., 2021). However, the loading capacity using this approach is low, generally less than 10%, and significantly increases the metabolic burden on the body through the additional adjuvants. In addition, the weak connections between the molecules of the drug and the nanoparticles can lead to the untimely release of the drug after intravenous administration. Direct chemical bonding of the drug with the amphiphilic polymer creates a drug delivery system with high drug loading that has excellent stability in the blood circulation and can inhibit the premature release of the drug (Deng & Liu, 2020; Liu et al., 2021). Such polymeric prodrug delivery systems can not only improve drug loading significantly but can also precisely control drug loading and pharmacokinetics through chemical reaction. The construction of endogenous stimulus-sensitive polymeric prodrug nanoparticles allows the development of on-demand or triggered drug delivery.

Chemical groups sensitive to external signals (temperature, light, magnetic field, and ultrasonic wave) and endogenous signals [pH, reduction, enzyme, and reactive oxygen species (ROS)] have been extensively exploited in the design of polymeric prodrug delivery systems for triggered drug release (Li et al., 2015; Chen et al., 2018). The pH gradient in the tumor microenvironment (e.g., tumor tissue is around 6.5, endosomes are around 5.5, lysosomes are around 5.0) can act as an endogenous stimulus for controlling the drug release in tumor cells (Dai et al., 2019), whereas acidity is a specific feature of the tumor microenvironment; acid-sensitive coupling linkages are often used to construct polymeric prodrug nanoparticles with efficient transformation in tumors. It is reported that there is a significant difference in the concentration of reduced glutathione (GSH) between the microenvironment inside and outside tumor cells. The differences in GSH between intracellular (2–10 mM) and extracellular environments (2–20 μM) of tumor cells, which are often exploited for reduction-sensitive drug delivery system. Therefore, the prodrug with disulfide bonds releases the parent drug effectively in tumor cells and improves the drug utilization (Wang et al., 2020; Zuo et al., 2020). The design and study of enzyme-triggered polymeric prodrugs have potential clinical applications (Srinivasan et al., 2021; Tan et al., 2021). Phospholipases, oxidoreductases, and proteases overexpressed by tumor cells can act as selective triggers for enzyme-sensitive drug delivery vehicles. Nanodrug delivery systems with reactive oxygen species (ROS) sensitivity can also promote the drug release as the level of ROS in tumor cells is ten times higher than that in normal cells (Saravanakumar et al., 2017; Deng et al., 2020; Hong et al., 2021; Tan et al., 2021). For example, the concentration of H_2_O_2_ is 0.001–0.7 μM in healthy cells, while it is 10–100 μM in tumor cells (Li C. et al., 2021). ROS-sensitive chemical groups such as alkylene sulfides, borate esters, and thioketals (TKs) have been widely exploited to construct efficient stimulus-sensitive drug delivery systems for tumor therapy. However, due to the heterogeneity of tumor cells, the concentration of endogenous ROS is too low to trigger rapid drug release, and few ROS-sensitive drug delivery systems show sufficient sensitivity to tumor cells. Therefore, a ROS-sensitive drug delivery system with the ability to generate ROS would be a promising strategy that could improve selectivity and accelerate drug release to enhance the efficacy of tumor therapy. The construction of the stimulus-sensitive polymeric prodrug will improve the stability and drug loading of PTX, enable its controlled release at the tumor site, enhance the drug utilization, and reduce side effects.

**FIGURE 1:**
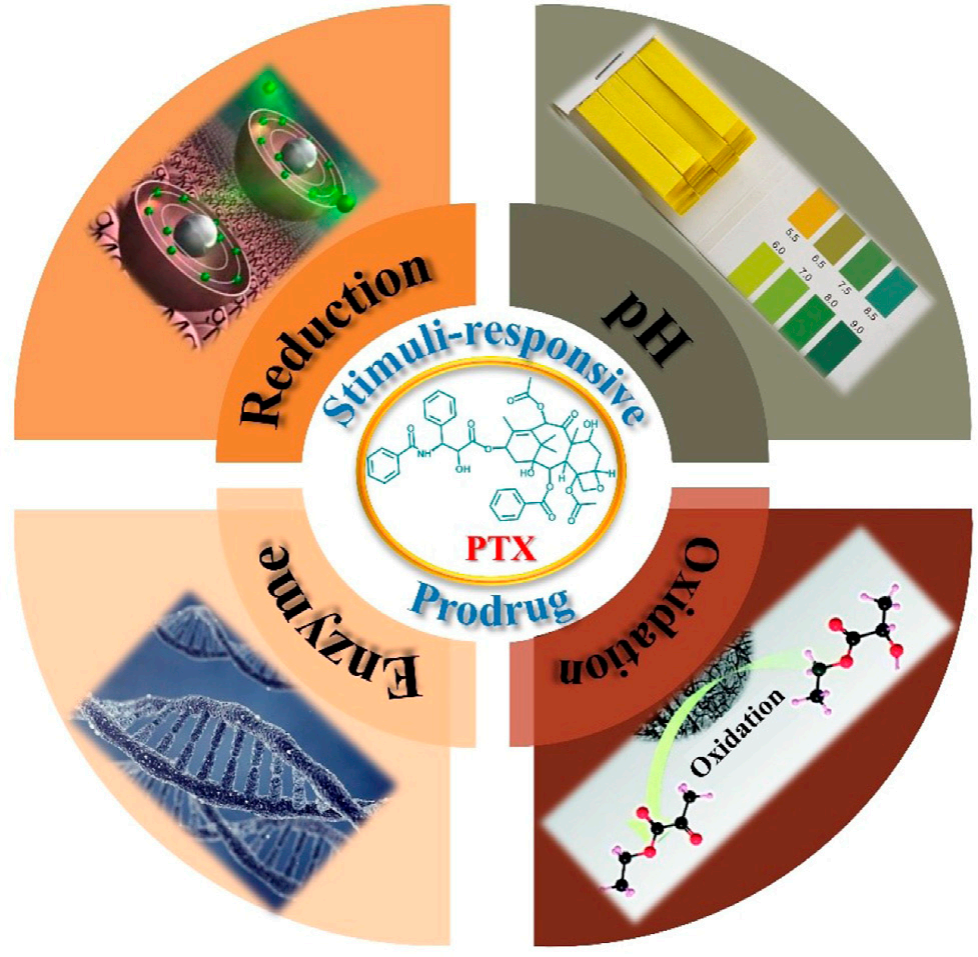
Four types of polymer-PTX prodrug stimuli-sensitives.

As far as we know, much of the pioneering work on stimulus-sensitive polymeric PTX prodrug nanomedicine has not been well summarized. As shown in Figure 1, research on this subject can be categorized by the four different types of polymer-PTX prodrug stimuli: 1) pH-sensitive polymer-PTX prodrug, 2) reduction-sensitive polymer-PTX prodrug, 3) enzyme-sensitive polymer-PTX prodrug, and 4) ROS-sensitive polymer-PTX prodrug. This review focuses on the design, synthesis, and biomedical applications of stimulus-sensitive PTX polymer prodrugs and the latest research progress. Finally, the future development directions and prospects of this field were briefly discussed.

## pH-Sensitive Polymer-PTX Prodrug

Compared with that of normal tissue, the tumor microenvironment is highly acidic, which is due to the high metabolic activity of tumor cells. Therefore, the development of pH-sensitive polymer-PTX prodrugs in which drug release is triggered by the acidity of tumor cells has attracted extensive attention. To achieve this, acid-sensitive organoleptic groups, such as acetals and acyclic ketals, are typically built into polymeric prodrug delivery systems to cause the release of the drug when the delivery system encounters an acidic microenvironment. For instance, Zhai et al. developed pH-sensitive polymer-PTX prodrug micelles by grafting PTX onto the main chain of poly (ethylene glycol) (PEG)-polycarbonate through an acetal-linker to accelerate drug release in tumor cells. The drug loading of PTX reached up to 33% (Zhou et al., 2020a). mPEG-PCL-Ace-PTX is a pH-sensitive PTX prodrug micelle with the PTX content as high as 23.5 wt%. This was constructed using functionalized poly (ethyleneglycol)-poly (ε-caprolactone) (mPEG-PCL) diblock polymer with an acid-cleavable acetal (Ace) linkage (Zhai Y. et al., 2017). Another pH-sensitive amphiphilic polymeric prodrug, PEG-acetal-PTX, has been constructed for the inhibition of tumor cell proliferation.

This self-assembled prodrug has excellent stability with drug loading up to 60.3% (Huang et al., 2018). Acyclic ketals are generally more acid-sensitive than acetals, so the development of pH-sensitive polymeric prodrug micelles based on acyclic ketal–coupled linkers are more desirable (Mu et al., 2020). Guo et al. constructed acyclic-ketal–based acid-sensitive PTX prodrug micelles using PEG with different lengths. They found that the length of PEG affects the hydrolysis kinetics, pharmacokinetics, biodistribution, and antitumor activity of the prodrug nanoparticles (Mu et al., 2020).

## Reduction-Sensitive Polymer-PTX Prodrug

**FIGURE 2:**
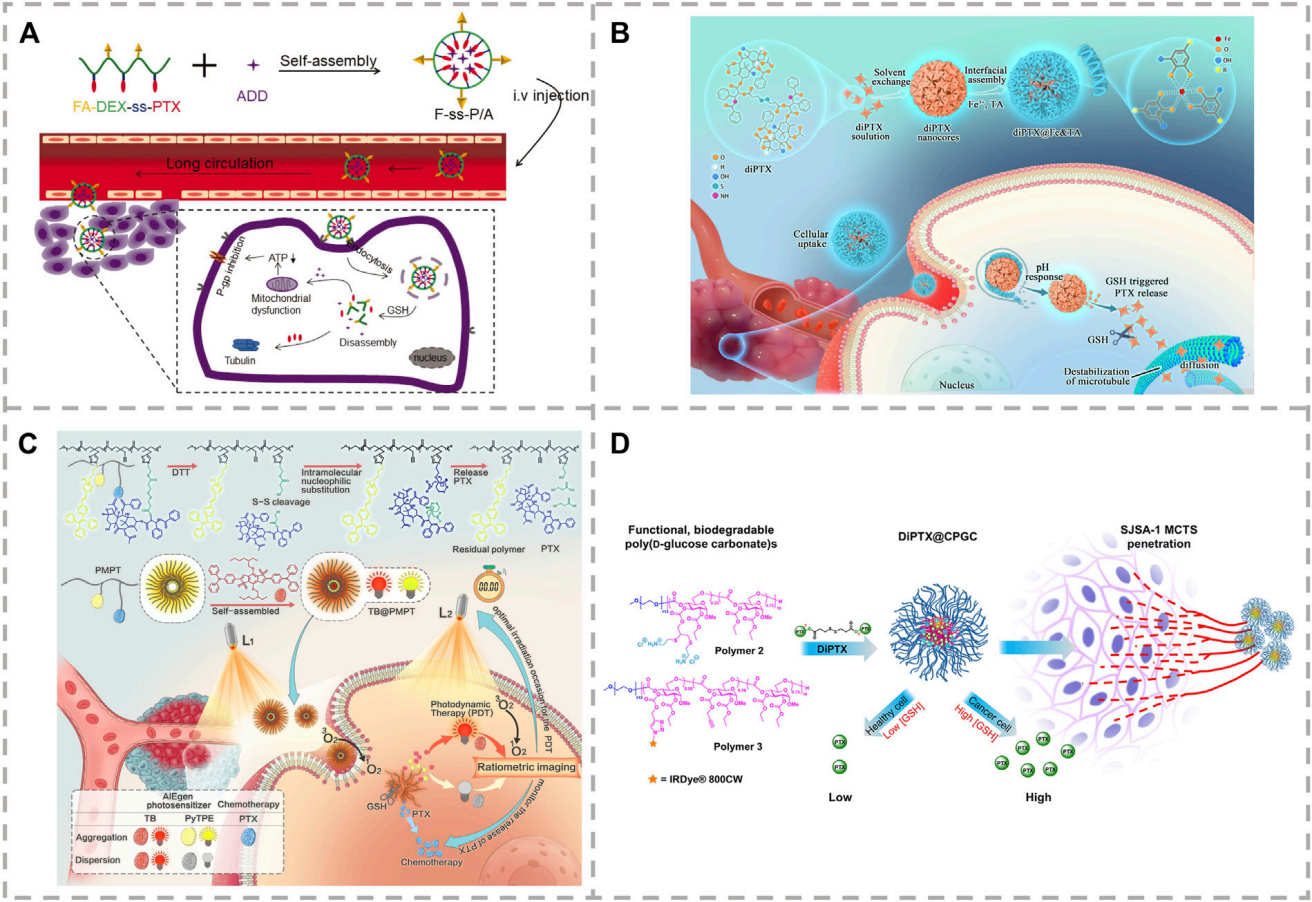
**(A)** Release and mechanism of the self-assembly of F-ss-P/A micelles (Chen et al., 2020). **(B)** Synthesis and mechanism of PTX release from polymeric prodrug of TB@PMPT (Yi et al., 2021). **(C)** Procedure, intracellular pH, and reduction-sensitive of programmed drug release from dimeric prodrug nanoparticles (Yi et al., 2022). **(D)** Release of PTX from the poly (ethylene glycol) (PEG)-protected reduction-sensitive dimeric PTX (diPTX)-loaded cationic poly (D-glucose carbonate) micelle (diPTX@CPGC) (Su et al., 2018).

Due to the high expression of GSH in tumor cells, polymeric prodrugs with reduction-sensitive properties can achieve controlled intracellular drug release. Li et al. constructed a folate-targeted reduction-sensitive polymeric prodrug micelle by coupling PTX with dextrin through disulfide bond and embedded mitochondrial inhibitor of adjudin to overcome any multi-drug resistance of tumor cells (Figure 2A) (Chen et al., 2020). Liu et al. demonstrated that the reduction-sensitive PTX prodrug modified with maleimide functional groups was able to rapidly bind the circulating albumin immediately after intravenous administration (Lou et al., 2021). The albumin-bound PTX–maleimide prodrug nanoparticles exhibited longer circulation time and excellent anti-tumor efficacy *in vivo*. This disulfide-bridged prodrug exhibited selective cytotoxicity toward tumor cells and enhanced tumor suppression in BALB/C mice bearing 4T1 tumor. Xie et al. reported a reduction-sensitive prodrug of PTX (PTX-S-BDP) by linking PTX and BODIPY (BDP-OH) using a reduction-sensitive linker for fluorescence imaging–guided chemotherapy (Xia et al., 2021). PTX-S-BDP nanoparticles display excellent cellular imaging and good cellular selectivity, with free PTX reserving its cytotoxic effects for cancerous cells. Other studies have endeavored to produce synergistic therapeutic effects by combining two or more agents with different target signaling pathways (Zhou et al., 2020b). Gu et al. synthesized an amphiphilic reduction-sensitive prodrug of PTX-SS-TMP to deliver both PTX and the antiangiogenic agent of tetramethylpyrazine (TMP) to realize the synergistic treatment of A2780 tumor-bearing mice (Zou et al., 2021).

In addition, chemo-photodynamic therapy is an advanced treatment method that researchers are more concerned about (Yi et al., 2018). The combination of chemotherapeutic drugs and photosensitizers can improve therapeutic effects and reduce cytotoxic effects. In the development of this drug, an amphiphilic polymeric prodrug was used as a carrier of the photosensitizer needed for the combined treatment under light irradiation. A further advantage of this synthesis is that the ROS produced by photodynamic therapy can induce lipid peroxidation, increasing the permeability of the cell membrane and enhancing the intracellular internalization of PTX (Figure 2B) (Yi et al., 2021). Dimeric prodrugs can achieve high drug loading and inhibit premature drug leakage, so this strategy based on dimeric prodrugs is of great significance for the design of novel nanomedicine (Pei et al., 2018). The nanoparticles, diPTX@Fe&TA (Figure 2C), and diPTX@CPGC Figure 2D were prepared through co-precipitation of the dimeric prodrug, PTX-SS-PTX (diPTX), with the metal-phenolic network of Fe and tannic acid (Fe&TA) or cationic poly (D-glucose carbonate) (CPGC), respectively (Su et al., 2018; Yi et al., 2022). The drug loading of diPTX was 24.7 and 40% for diPTX@Fe&TA and diPTX@CPGC, respectively. The premature release of PTX and diPTX in the physiological environment was greatly inhibited. Compared with intravenous injection, oral administration is a more preferred choice for PTX administration, which is mainly due to the more convenient, safe, and flexible administration (Du et al., 2020; Gao Y. et al., 2021). The prodrugs, PTX-Cys, PTX-SS-COOH, and PTX-SS-Val were prepared by introducing amino, carboxyl, and valine into the disulfide bond of PTX to improve the solubility and oral bioavailability (Li Y. et al., 2021). Of these three, it was found that PTX-SS-Val can effectively improve the oral bioavailability of PTX.

## Enzyme-Sensitive Polymer-PTX Prodrug

**FIGURE 3:**
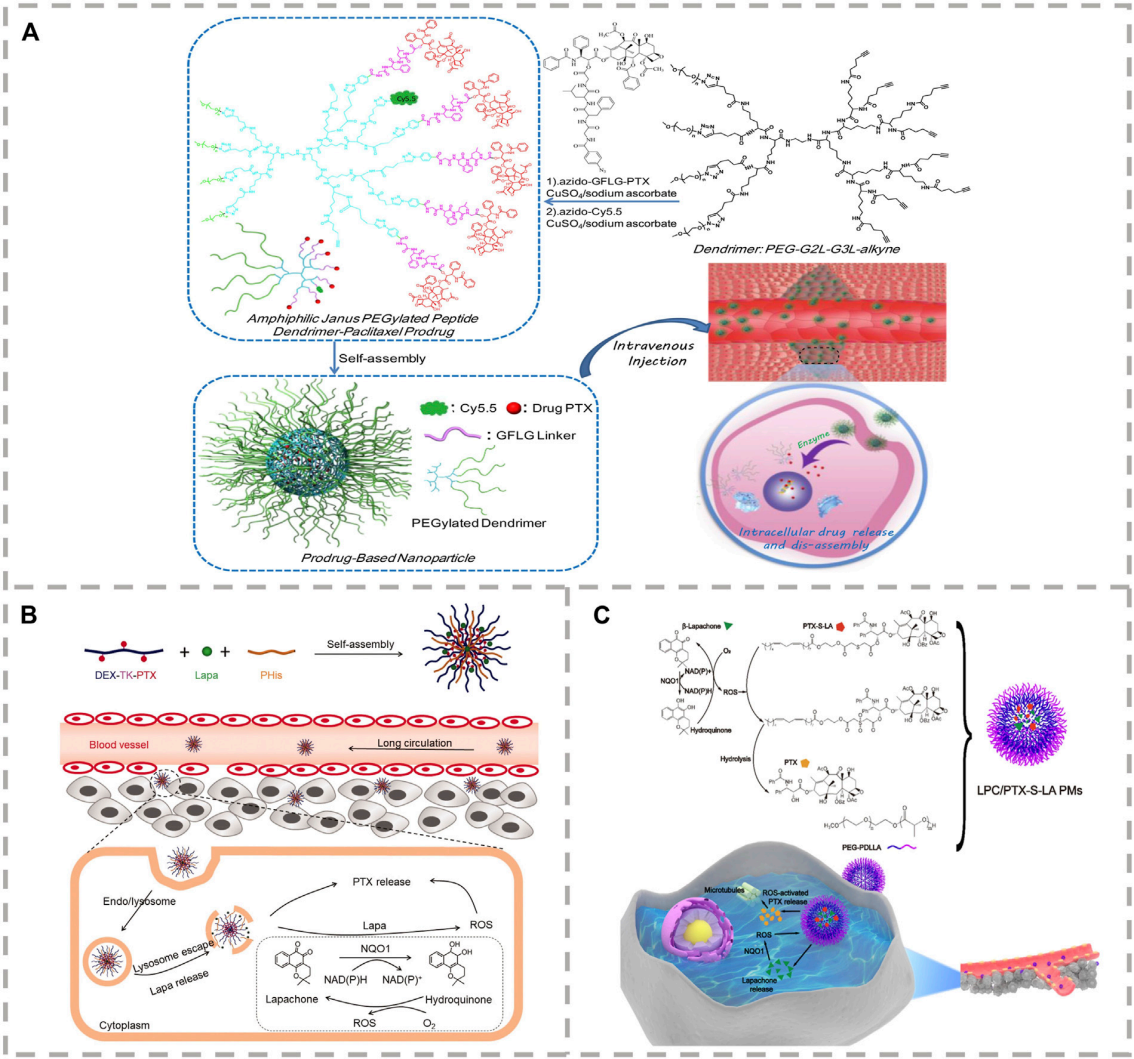
**(A)** Structure and synthesis of enzyme-sensitive amphiphilic Janus PEGylated dendrimer-GFLG-PTX prodrug and its released PTX from prodrug through intracellular enzymolysis after endocytosis (Li et al., 2017). **(B)** A DEX-TK-PTX–based self-accelerating drug release nanodrug delivery system (PLP-NPs) with pH/ROS cascade response was constructed for the treatment of multidrug resistant colon cancer (Chang et al., 2020). **(C)** Self-strengthened bioactivating prodrug-based NPs are fabricated *via* co-encapsulation of ROS-sensitive thioether-linked linoleic acidpaclitaxel conjugates (PTX-S-LA) and β-lapachone (LPC) into polymeric micelles (PMs) (Wang K. et al., 2019).

Enzymes have been used extensively as prodrug release triggers for stimulus-sensitive materials due to their high selectivity toward tumor cells (Pei et al., 2021; Zeng et al., 2021). For example, cathepsin B is overexpressed in the cells of various types of tumors, which acts as a lysosomal cysteine protease and can cleave proteins containing an oligopeptide glycylphenylalanylleucylglycine (Gly-Phe-Leu-Gly, GFLG) linker (Cheng et al., 2020; Jin et al., 2020). The GFLG linker between the drug and the polymer in the construction of cathepsin B–based enzyme-sensitive drug carriers is shown in Figure 3A. Luo et al. have constructed a series of amphiphilic polymer-PTX prodrugs using the enzyme-sensitive GFLG. They also used the polymeric prodrugs as carriers to load other functional drugs for real-time monitoring, tumor diagnosis, and therapy (Li et al., 2017; Wang B. et al., 2019; Luo et al., 2021; Tan et al., 2021). For example, the enzyme-sensitive amphiphilic prodrug, Janus PEGylated dendrimer-GFLG-PTX, was prepared by conjugating the GFLG-PTX moiety to the PEGylated peptide dendrimer through click reaction (Li et al., 2017). *In vitro* experiments found that the cytotoxicity of the polymer prodrug on normal cells is far lower than that of free PTX, and it was able to effectively induce the apoptosis of breast cancer cells. A diagnostic and therapeutic platform was created using a PTX saccharide-based prodrug (pGAEMA-PTX-Ppa-Gd polymer) containing enzyme-sensitive GFLG oligopeptide (Wang B. et al., 2019). The *T*_1_ contrast agent of Gadolinium-tetraazacyclododecanetetraacetic (Gd-DOTA) and the NIR fluorescent molecule pheophorbide a (Ppa) were conjugated to the polymer for cancer diagnosis, treatment, and real-time monitoring. In addition, an amphiphilic block copolymer prodrug, poly [oligo (ethylene glycol) methyl ether methacry-late] (polyOEGMA)-functionalized PTX, containing cathepsin B–sensitive GFLG oligopeptide linker between the PTX and the polymer backbone was constructed (Tan et al., 2021). This was then used as a carrier to load the photosensitizer Ce6 for combined chemo-photodynamic therapy. This effectively inhibited the growth of bladder cancer patient-derived tumor xenograft models through the photochemical internalization effect. Delivering the therapeutic agent to the site of the malignancy and activating deep tissue. A polyOEGMA-functionalized dendritic polymer-PTX prodrug with a short peptide GFLG was prepared to encapsulate an imidazole derivative with high energy-transfer efficiency and synergistically enhance the effects of two-photon photodynamic therapy effect for the synergistic effect on inhibition of tumor growth in 4T1 xenograft mice by combined chemo-photodynamic therapy with increased penetration depth (Luo et al., 2021).

## ROS-Sensitive Polymer-PTX Prodrug

It is well known that benzylboronic ester can trigger an elimination response through the high levels of ROS in tumor cells, while the benzylboronic ester linkers have demonstrated excellent stability during blood circulation. For example, Shen et al. constructed the novel ROS-sensitive prodrug micelles based on *p*-(borate ester)benzyl by introducing the benzylboronic ester linker between the PTX and the PEG (PEG-B-PTX) (Dong et al., 2020). The loaded quinone was able to deplete intracellular GSH with the PTX and effectively inhibit the growth of tumor cells. The TK group has excellent stability in a normal physiological environment; the high level of ROS causes the group to cleave to generate acetone and two other thiol-containing fragments. However, the level of ROS expression in tumor cells is insufficient to trigger drug release completely. A promising strategy to address this is the development of drug delivery systems able to provoke intracellular ROS generation. Menadione (VK3) is a quinoid natural molecule with anti-tumor effects in breast, lung, prostate, and liver cancers. VK3 can catalyze ROS production through the specific overexpression of NAD(P)H:quinone oxidoreductase-1 (NQO1) in tumor cells and specifically improve the intracellular ROS level (Yang et al., 2018). Lu et al. developed the dual-responsive pH/ROS polymeric prodrug of PEG-b-P(LL-g-TK-PTX)-(LL-g-DMA) by conjugating the ROS-sensitive PTX prodrug (PTX-TK) and 2,3-dimethylmaleic anhydride (DMA) into the main chain of the amphiphilic polymer, PEG-b-PLL (Xu et al., 2020). Then, they encapsulated VK3 into the core of PEG-b-P((LL-g-TK-PTX)-(LL-g-DMA)) micelles to construct a self-amplifying drug delivery system with charge reversal capability. High levels of NQO1 in tumor cells catalyze the released VK3 to produce ROS, resulting in the amplified release of PTX and reduced side effects. To overcome insufficient intracellular release of nanodrugs and multi-drug resistance (MDR) of chemotherapeutics in the treatment of colon cancer, a ROS-sensitive thioketal (TK) bond combined PTX and dextran (DEX) to synthesize the prodrug DEX-TK-PTX. Then, pH-sensitive poly (L-histidine) and beta-lapachone were loaded into prodrug micelles, creating a self-accelerating drug release nanodrug delivery system (PLP-NPs) with pH/ROS cascade response for the treatment of multidrug-resistant colon cancer (Chang et al., 2020). PLP-NPs can increase the intracellular ROS level and drug concentration and consume the level of ATP in multidrug-resistant tumor cells (Figure 3B). To increase the deep penetration ability and intracellular release of nanodrugs at the site of malignancy, Sun et al. synthesized three polymers, the ROS-sensitive (methoxyl poly (ethylene glycol)-thioketal-paclitaxel (mPEG-TK-PTX)), iRGD-PEG-TK-PTX, and the pH-sensitive polymer octadecylaminepoly (aspartate-1-(3-aminopropyl) imidazole) (OA-P(Asp-API)) (Li et al., 2020). The three amphiphilic polymers were then used as carriers to encapsulate the ROS generation agent β-Lapachone (LAP) and construct multifunctional polymeric micelles (RLPA-NPs). RLPA-NPs can penetrate the tumor tissue through iRGD, release LAP in lysosomes, and amplify the release of PTX, ultimately improving the therapeutic effect. Ether is a relatively stable substance, while thioether is easily oxidized into sulphone and sulfoxide, resulting in a strong increase in its hydrophilicity and in the hydrophilic–hydrophobic ratio of polymers. Sun et al. synthesized the ROS-sensitive prodrug of thioether-linked PTX-linoleic acid conjugates (PTX-S-LA) (Figure 3C) (Wang K. et al., 2019), and combined it with a ROS generating agent to create a self-strengthening bioactive prodrug nanosystem with powerful anticancer effects. ROS produced by photosensitizers under light can induce tumor cell death and trigger the cleavage of thioether. PTX and the photosensitizer pyropheophytin a (PPa) were linked by a ROS-sensitive thioether linkage to construct a “two-in-one dimer” that was both carrier and cargo (Luo et al., 2019). Under laser irradiation, the overproduction of endogenous ROS and the ROS produced by the PPa synergistically triggered the release of PTX.

## Summary and Future Perspectives

The presence of the polymer shell stabilizes the polymer-based drug delivery system. Thus, they have the advantages of controlled PTX release, reduced dosage, and reduced systemic side effects. However, polymer-based drug delivery systems have some limitations. Organic solvents or surfactants, such as those often used in the preparation of polymer nanoparticles, can disrupt biological membranes and have significant interaction with certain proteins. At the cellular level, polymer-based nanodrug delivery systems are not as biocompatible as liposomes. The structure of polymeric nanoparticles is unstable, and the encapsulation of a large amount of PTX can lead to colloidal transformation. There is a main disadvantage that polymer nanoparticles lack selectivity toward tumor cells. Although nanoparticles can passively target solid tumors through the EPR effect, the majority of polymer prodrugs nanoparticles with the size range from 100 to 500 nm will still be cleared by the reticuloendothelial system. Active targeting can achieve specific uptake of tumor cells, further improve drug utilization, and reduce side effects. Polymers are generally modified targeting functional groups by chemical reaction to achieve active targeting.

From the reviewed studies, it can be seen that a number of biocompatible polymers have been used and characterized for PTX prodrug formulation (Table 1). Amphiphilic polymer is the most widely studied material, but in fact, proteins are biodegradable, nonantigenic, and metabolizable, which provide a variety of possibilities for drug delivery and is a promising material for adjuvants. Many types of nanoscale drug delivery systems have been used to deliver PTX, including micelles, polysomes, liposomes, and fibers. From these studies, it can be concluded that tumor microenvironment stimulus-responsive polymeric prodrugs not only show outstanding stability and solubility but also have a controlled PTX release profile and biocompatibility. Therefore, prodrug nanoparticles hold exciting promise as a potential PTX delivery tool.

**TABLE 1:** Summary of representative systems for PTX prodrugs.

Type	Sensitive group	Material	Cancer type	References
pH	Acetal	APPMs	A549	Zhai et al. (2020)
		mPEG-PCL-Ace-PTX_5_	MCF-7	Zhai Yinglei et al. (2017)
		PEG-acetal-PTX (PAP)	HeLa and MDA-MB-231	Huang et al. (2018)
	Acyclic-ketal	PEGylated acetone- acyclic-ketal-PTX (PKPs)	A2780 tumor-bearing mice	Mu et al. (2020)
Reduction	Thioether and disulfide	PSMAL and PSSMAL	4T1 tumor-bearing mice	Lou et al. (2021)
	Thioether	PTX-S-BDP.	HeLa and L929	Xia et al. (2021)
	Disulfide	PTX-ss-TMP	A2780 tumor-bearing mice	Zou et al. (2021)
		TB@PMP	HeLa tumor-bearing mice	Yi et al. (2018)
		TB@PMPT	HeLa tumor-bearing mice	Yi et al. (2021)
		diPTX@Fe&TA	HeLa tumor-bearing mice	Yi et al. (2022)
		diPTX@CPGC	NOD/SCID IL2-R-gamma mice	Su et al. (2018)
		PTX-SS-CIT	4T1 tumor-bearing mice	Gao Yuanyuan et al. (2021)
		PTX-SS-Val, PTX-SS-COOH	MCF-7	Li Yanlin et al. (2021)
		FA-DEX-ss-PTX	HCT-8/PTX tumor-bearing mice	Chen et al. (2020)
	Azo	Ce6/PTX2-Azo	HeLa tumor-bearing mice	Zhou et al. (2020b)
Enzyme	GFLG	PEGylated dendrimer-GFLG-PTX	4T1 tumor-bearing mice	Li et al. (2017)
		pGAEMA-PTX-Ppa-Gd	4T1 tumor-bearing mice	Wang Bing et al. (2019)
		dendritic- [(GFLG-polyPTX)-block-polyOEGMA]	4T1 tumor-bearing mice	Luo et al. (2021)
		poly (OEGMA)-PTX@Ce6	PDX tumor bearing mice	Tan et al. (2021)
	Borate ester)benzyl	PEG-B-PTX	MCF-7 tumor-bearing mice	Dong et al. (2020)
ROS	Thioketal	RBC(M(TPC-PTX))	HeLa tumor-bearing mice	Pei et al. (2018)
		PEG-b-P(LL-g-TK-PTX)-(LL-g-DMA)	PC-3 tumor-bearing mice	Xu et al. (2020)
		DEX-TK-PTX	HCT-8/PTX tumor-bearing mice	Chang et al. (2020)
		RLPA-NPs	4T1 cells tumor-bearing mice	Li et al. (2020)
	Thioether	LPC/PTX-S-LA PMs	4T1 tumor-bearing mice	Wang Kaiyuan et al. (2019)
		PPa-S-PTX	KB and 4T1 tumor-bearing mice	Luo et al. (2019)

## Conclusion

The application of nanotechnology to cancer treatment has led to several breakthroughs previously and continues to flourish as a key component of the health care system. Abraxane is the only nano-PTX formulation approved by the FDA and EMEA for the treatment of cancer, and is the most successful PTX formulation in clinical research. In this review, we have focused on amphiphilic polymer-PTX prodrug delivery systems and their ability to provide stimulus-sensitive therapeutic nanoplatforms for intracellular on-demand drug release. Polymeric prodrug nanoparticles can not only greatly improve the drug loading and solubility of PTX but can reduce its toxicity. In the future development of more promising nano-prodrug PTX formulations, some challenges will need to be overcome. Among them, there is the need to improve the efficiency of drug enrichment and the specificity of its release at the lesion site.

Despite this, the efficacy remains low for the prodrugs developed thus far. This may be due to the multilevel and complex biological issues inherent in attempting to eliminate cancerous tissues with minimum harm to the human body. Achieving this end requires targeted nanocarriers with strong specificity. To sum up, the research discussed in this review has made advances but there are still limitations to overcome. We hope that this overview will provide some ideas for future nano-prodrug delivery systems.
